# Artificial intelligence and digital health equity: a post-pandemic evidence synthesis and implementation safeguards framework

**DOI:** 10.3389/fdgth.2026.1785700

**Published:** 2026-05-18

**Authors:** Albert Nii Noi Okwei

**Affiliations:** L. Douglas Wilder School of Government and Public Affairs, Virginia Commonwealth University, Richmond, VA, United States

**Keywords:** algorithmic bias, artificial intelligence, COVID-19, digital divide, digital health equity, health disparities, health policy, telehealth

## Abstract

**Introduction:**

The rapid expansion of AI-enabled digital health after COVID-19 created new possibilities for extending care while raising concerns about unequal access, subgroup underperformance, and weak accountability. These effects remain difficult to interpret because telehealth infrastructure, predictive analytics, clinical decision support, and generative AI operate through different equity mechanisms.

**Methods:**

A transparent narrative evidence synthesis was conducted using peer-reviewed literature and selected governance documents. Structured searches of PubMed, Scopus, Web of Science, and IEEE Xplore were refreshed on March 5, 2026, for English-language sources published between January 1, 2020, and December 31, 2025. A second reviewer independently assessed a sample of 12 full-text inclusion and exclusion decisions using the same pre-specified criteria, with 100% agreement. The final corpus included 29 sources.

**Results:**

Evidence was strongest for digital access barriers, subgroup underperformance, and implementation-related governance concerns. AI-assisted screening and digitally mediated remote support showed conditional benefits in targeted settings when high-touch support was built into implementation. Evidence on generative AI and language-support tools was comparatively smaller, newer, and concentrated in representational-bias applications, with limited evidence of sustained downstream equity gains.

**Conclusion:**

AI-enabled digital health may reduce selected barriers to care when supported by equitable access conditions, subgroup validation, accountable governance, and implementation oversight. Equity gains were conditional rather than automatic, and evidentiary depth varied substantially across modalities. The review distills recurring patterns into a synthesis-derived operational roadmap for procurement, validation, implementation, and post-deployment monitoring.

## Introduction

The post-2020 acceleration of digital health changed the conditions under which healthcare access is organized, experienced, and governed. Patient portals, telehealth platforms, remote monitoring systems, and algorithm-supported workflows now influence who can reach care, how quickly care is delivered, and how clinical attention is prioritized. The pandemic-era expansion of digital care intensified longstanding concerns about racialized, socioeconomic, geographic, linguistic, and age-based inequities in healthcare access and outcomes (1, 2).

Artificial intelligence has become increasingly embedded within this digital health ecosystem, but it is not a single type of intervention. In practice, the term covers several distinct categories: telehealth infrastructure, algorithmic AI systems, predictive analytics, clinical decision support, and generative AI or language tools. These categories operate through different equity mechanisms and should not be treated as interchangeable. Telehealth infrastructure primarily affects access by changing where and how care is delivered. Predictive analytics and clinical decision support shape resource allocation by determining which patients are flagged, prioritized, or routed for intervention. Generative AI and language tools may affect usability, communication, and documentation, but the empirical evidence on their equity effects remains substantially thinner than the evidence on screening or remote care.

Several recent reviews have advanced this field, but along different analytical lines. Berdahl et al. (3) mapped AI-related health equity issues and mitigation strategies across lifecycle stages. Petretto et al. (4) synthesized telemedicine, e-health, and digital health equity barriers and interventions. Ghanem et al. (5) examined public health AI and emphasized governance, accessibility, and implementation concerns. Kim and Backonja (6) reviewed digital health equity frameworks and showed that the field contains many partial models but no single comprehensive framework. What remains less well synthesized is the post-2020 implementation-facing intersection of these literatures: how distinct AI-enabled digital health modalities have operated across access, adoption, subgroup performance, and governance in real or near-real health system settings, and where evidentiary depth remains uneven across modalities.

This review addresses that gap through a transparent narrative evidence synthesis focused on the post-pandemic period. The central question is not whether AI is inherently equity-promoting or inequity-producing. A more useful implementation question is: under what conditions do AI-enabled digital health tools reduce selected barriers to care, under what conditions do they reproduce or intensify inequity, and where does the current evidence base remain stronger or weaker across modalities? The review therefore distinguishes the mechanisms through which equity effects are produced: access conditions, adoption conditions, model-performance conditions, and governance conditions.

The analysis was informed by three complementary lenses applied in layered rather than interchangeable fashion. Digital Divide Theory was used to interpret access, connectivity, literacy, language accessibility, disability accommodation, and organizational infrastructure barriers (2, 7). The Technology Acceptance Model was used to interpret adoption-related dynamics, including perceived usefulness, ease of use, trust, and workflow fit (8). Critical Public Health was used to examine how data representation, institutional power, procurement choices, and accountability structures shape whose needs are centered, whose risks are externalized, and whose outcomes are optimized (9, 10). These lenses support a multi-level reading of equity effects across access conditions, adoption conditions, model-performance conditions, and governance conditions.

## Methods

### Study design

This study used a transparent narrative evidence synthesis rather than a systematic review or meta-analysis because the evidence base was heterogeneous in design, intervention type, and outcome structure. A narrative synthesis was better suited to integrating empirical findings with implementation and governance insights while maintaining transparency about search and selection procedures.

### Search strategy

A concept-block search strategy was used to identify literature at the intersection of artificial intelligence, digital health, and health equity. Searches were conducted across PubMed, Scopus, Web of Science, and IEEE Xplore and were refreshed on March 5, 2026. Eligibility was restricted to English-language sources published between January 1, 2020, and December 31, 2025. The search combined four concept blocks: (1) AI and analytics terms, including “artificial intelligence,” “machine learning,” “predictive analytics,” “clinical decision support,” “natural language processing,” “large language model,” and “generative AI”; (2) digital health delivery terms, including “digital health,” “telehealth,” “telemedicine,” “remote patient monitoring,” and “mobile health”; (3) equity terms, including “health equity,” “health disparities,” inequity, underserved, marginalized, vulnerable, “digital divide,” “social determinants of health,” and “limited English proficiency”; and (4) healthcare setting terms, including healthcare, “public health,” clinic, hospital, patient, screening, and implementation. Database-specific syntax was adapted for each platform. Targeted citation chaining and selected governance-source searches supplemented database retrieval.

### Operational definitions

To address definitional ambiguity, the following categories were used consistently. Telehealth infrastructure refers to digital channels used to deliver care, including video visits, telephone visits, messaging, portals, and remote monitoring interfaces. Algorithmic AI systems refers to computational systems in which algorithmic processing materially shapes classification, prediction, triage, or automated interpretation. Predictive analytics refers to models used to estimate risk, identify likely events, or support forecasting. Clinical decision support refers to tools that provide clinicians or care teams with recommendations, risk scores, or alerts influencing diagnosis, referral, escalation, or treatment. Generative AI or language tools refers to systems such as large language models or generative image tools used for summarization, translation, documentation, or patient communication. These categories were analytically linked to four equity mechanisms: access, usability, decision support, and resource allocation.

### Inclusion and exclusion criteria

Sources were included if they met all of the following conditions: (1) published between January 1, 2020, and December 31, 2025, or directly relevant as limited foundational context for post-2020 implementation and governance; (2) located in a healthcare or public health setting; (3) addressed AI-enabled digital health or a digital health modality directly relevant to AI deployment conditions; (4) included at least one equity-relevant element such as access barriers, subgroup performance, bias, adoption disparities, governance, or implementation for underserved populations; and (5) published in English. Sources were excluded if they were purely technical performance papers with no healthcare implementation context, reported model development only without a clear health equity implication, were opinion pieces without substantive framework or implementation content, or focused exclusively on pre-2020 contexts unless retained as limited foundational background.

### Screening and selection

Screening was conducted in two stages: title and abstract screening, followed by full-text assessment. Potentially relevant records were retained at the first stage when equity relevance was plausible but unclear. To assess screening consistency, a second reviewer independently evaluated a sample of 12 full-text inclusion and exclusion decisions, representing 15% of the 80 full-text assessments, using the same pre-specified criteria. Agreement was 100%, confirming that the criteria were applied consistently across both stages. Because the corpus was primarily screened by a single author, residual selection bias remains possible, particularly toward studies using explicit equity language and away from technically oriented validation or fast-moving generative AI implementation studies whose subgroup implications may be less visible in titles or abstracts.

### Deduplication

Duplicates were removed manually using DOI, article title, first author, journal, and year as matching fields. Where records appeared in multiple databases with minor formatting differences, they were treated as duplicates if the DOI or bibliographic identity matched.

### Corpus composition

The final evidence corpus included 29 sources: 12 empirical studies, 7 reviews, 4 framework papers, and 6 governance or policy analyses. Empirical studies were prioritized when presenting claims about equity-relevant outcomes. Reviews, framework papers, and governance analyses were used primarily to contextualize recurring implementation patterns and accountability challenges. References cited outside this 29-source corpus were used selectively as adjacent contextual literature to clarify conceptual distinctions, situate findings within broader clinical and algorithmic equity debates, or interpret emerging evidence domains not strongly represented in the final corpus. A full listing of all 29 included sources is provided in Supplementary Table S1.

### Study selection process

Database searches identified 500 records (PubMed = 172; Scopus = 156; Web of Science = 121; IEEE Xplore = 51). Citation chaining and targeted governance-source searches identified an additional 23 records, yielding 523 total records. After manual deduplication, 141 duplicates were removed and 382 records remained for title and abstract screening. At that stage, 302 records were excluded. Eighty full-text sources were assessed for eligibility. Fifty-one full-text sources were excluded: no explicit equity, access, bias, or governance relevance (*n* = 19); purely technical model-development paper without implementation context (*n* = 14); commentary or opinion without substantive framework or implementation content (*n* = 9); outside the publication window (*n* = 5); not situated in healthcare or public health (*n* = 4). The final corpus included 29 sources.

### Data extraction and thematic organization

For each included source, the following information was extracted: study design, country or setting, population, digital or AI tool, reported outcome, and equity relevance. Sources were organized using a hybrid deductive-inductive thematic approach. Deductively, the analysis was structured around access conditions, adoption conditions, subgroup performance, and governance conditions. Inductively, patterns within the included studies refined the thematic organization. The manuscript uses the term thematically organized narrative evidence synthesis rather than claiming a formal line-by-line thematic synthesis procedure.

### Quality appraisal

A formal risk-of-bias tool was not applied because the corpus included diverse designs and purposes not readily comparable within a single appraisal instrument. Instead, claims were weighted according to transparency of methods, clarity of setting and sample, directness of the equity-relevant outcome, implementation relevance, and corroboration across multiple sources.

## Results

### Search outcome and corpus characteristics

The final corpus included 29 sources: 12 empirical studies, 7 reviews, 4 framework papers, and 6 governance or policy analyses. Empirical studies were concentrated in the United States and other higher-resource settings, with fewer primary studies from lower-resource contexts such as Dominica, Indonesia, Cameroon, and selected primary care settings in China. The evidence base was not uniform across modalities. Telehealth infrastructure and subgroup bias in predictive systems were supported by broader and more established literatures than generative AI and language-support applications, which remained comparatively newer and more concentrated in representational-bias and feasibility work.

### Telehealth infrastructure and digital readiness

Evidence on telehealth access and digital readiness was among the most consistently documented in the corpus, spanning empirical studies, reviews, and cross-sectional surveys across multiple underserved settings. The literature indicates that telehealth expansion did not, by itself, eliminate unequal access. Lam et al. (11) estimated that 13 million older adults in the United States might struggle to access telemedicine because of hearing, vision, communication, cognitive, or technology barriers. Adepoju et al. (12) found that 96% of surveyed adults in lower-income communities owned a smartphone, yet only about 30% had used remote patient monitoring; Black respondents were significantly less likely than White respondents to report remote patient monitoring use. Blount et al. (13) found near-universal clinician use of digital health tools alongside persistent patient-side barriers related to broadband access, cost, and fragmented interfaces. Taken together, these studies indicate that provider adoption and patient benefit should be treated as distinct outcomes.

### AI-assisted screening and remote clinical support

The strongest empirical evidence for equity-relevant benefit concerned AI-assisted screening and remote clinical support in targeted use cases, especially where specialist capacity or geographic reach was limited. Kemp et al. (14) reported that a smartphone-based diabetic retinopathy system in Dominica achieved sensitivity of 77.5% and specificity of 91.5% for referable disease in a real-world program involving 587 participants. Harsono et al. (15) found sensitivity of 80.0%, specificity of 96.4%, and accuracy of 93.8% for a smartphone-based AI application for cervical visual inspection in Indonesia across 199 women. Gu et al. (16) reported high accuracy and specificity for an AI fundus disease screening system across six primary healthcare settings in China with 4,795 participants. Nakisige et al. (17) found that an AI algorithm for cervical visual inspection performed comparably to healthcare workers and below expert performance, supporting its use as a decision-support adjunct. Mooney et al. (18) showed that automated symptom monitoring combined with clinician follow-up produced lower symptom burden than usual care, with non-White and lower-income participants showing larger symptom reductions. The most defensible synthesis is conditional: AI-assisted screening and digitally mediated remote support can extend capacity in targeted settings, but the strongest benefits were observed when implementation included structured human support.

### Predictive analytics and clinical decision support

Predictive analytics and clinical decision support carry particularly high equity stakes because they influence who is flagged, prioritized, or routed for intervention. Lo-Ciganic et al. (19) showed that incorporating human services and criminal justice data into an opioid overdose risk model for 237,259 Medicaid beneficiaries modestly improved predictive performance. Ingraham et al. (20) found that only 5 of 84 surgical clinical decision-support studies, or 6%, reported any equity analysis. Paulus and Kent (21) showed that average performance is not an adequate substitute for subgroup validity when predictive models inform consequential decisions.

Concerns about race-based algorithmic inequity extend well beyond any single clinical example. Hernandez-Boussard et al. (10) demonstrate that race-based clinical algorithms can produce unjust recommendations for minoritized patients by embedding race as a biological proxy, contributing to delayed diagnosis and reduced care access. Sjoding et al. (22) showed that pulse oximetry produces racially differential inaccuracies with measurable adverse clinical outcomes including elevated mortality risk from occult hypoxemia. Vyas et al. (23) documented that race-corrected estimated glomerular filtration rate equations systematically overestimate kidney function in Black patients, contributing to delays in transplant waitlist access. Coots et al. (24) further argue that fairness debates extend beyond explicit race variables to unequal decision rates, unequal error rates, and biased target variables. These findings suggest that race-based algorithmic inequity follows a cross-domain pattern, and that standardized subgroup validation and post-deployment equity auditing remain insufficiently institutionalized in routine clinical AI use.

### Generative AI and language tools

The evidence base on generative AI and language tools was substantially smaller, newer, and more uneven than the evidence on telehealth access barriers or subgroup bias in predictive systems. This section is offered as an agenda-setting review of emerging concerns rather than a synthesis of established findings; claims here carry lower evidentiary weight than those in prior sections. Peer-reviewed outcome evaluations of large language models used for multilingual patient communication or clinical translation were largely absent from the final corpus, consistent with broader literature confirming that this field remains primarily conceptual or pilot-based rather than outcome-driven (25, 26).

The clearest available evidence concerned representational bias. Fliorent et al. (27) reported that only 30% of identified dermatology AI programs included data on their use in skin of color. Joerg et al. (28) evaluated 4,000 AI-generated dermatologic images and found that 89.8% depicted light skin, only 10.2% depicted dark skin, and only 15% were identifiable as the intended dermatologic condition. Language-related inequities provide a clear rationale for effective language-support tools: Pandey et al. (29) found that limited English proficiency delayed access, weakened therapeutic relationships, and reduced treatment adherence; Ortega et al. (30) argued that language-appropriate care requires more than informal workarounds. However, current peer-reviewed outcome evidence on AI language tools remained insufficient to support broad claims that generative AI is already reducing health disparities at scale.

### Global and lower-resource implementation contexts

The corpus included fewer empirical studies from lower-resource settings, but those studies highlighted implementation conditions sometimes obscured in better-resourced systems. Kemp et al. (14), Harsono et al. (15), Nakisige et al. (17), and Sachdeva et al. (8) collectively suggest that AI-assisted screening may be most useful where specialist scarcity, travel burden, and limited diagnostic infrastructure constrain care. In Cameroon, acceptability depended on privacy, trust, explanation quality, and perceived usefulness rather than performance metrics alone (8). Lopez et al. (31) identified 40 implementation challenges and 89 recommendations spanning data quality, legal frameworks, infrastructure, and scalability. Alami et al. (32) argued that many AI applications are developed in high-income settings and transferred without adequate local validation. Townsend et al. (33) found that AI regulation across selected African countries remained fragmented and mediated through adjacent regimes rather than AI-specific law. The evidence suggests that AI-enabled digital health in lower-resource settings may support selected access goals only when implementation is context-specific, affordable, and institutionally supported.

## Discussion

### Main interpretation

The reviewed literature supports a restrained interpretation. AI-enabled digital health may reduce selected barriers to care when supported by equitable access conditions, user trust, subgroup validation, and accountable governance. Benefits were most evident in targeted screening and remote support contexts rather than in broad, system-wide disparity reduction. The equity implications of AI-enabled digital health are best understood as conditional rather than intrinsic: technology effects depended less on the mere presence of AI than on surrounding implementation conditions, including access infrastructure, language accommodation, subgroup validation, workflow fit, and governance capacity.

### Evidence depth across modalities

A central finding of this review is that evidentiary depth was not uniform across modalities. Digital access barriers, subgroup underperformance, and governance risks were supported by a broader and more established literature than the still-emerging evidence on generative AI and language-support applications. The strongest evidence concerned access barriers to telehealth, targeted AI-assisted screening in specialist-limited settings, and the persistent underdevelopment of subgroup auditing in predictive and decision-support tools. By contrast, the generative AI literature remained concentrated in a narrow set of representational-bias use cases and rarely extended to downstream patient outcome evaluation. The generative AI section should therefore be read as more agenda-setting and provisional than the sections on telehealth access or subgroup bias.

### Counterarguments and alternative interpretations

Alternative interpretations should be considered. Some digitally mediated inequities may partly reflect adoption-stage lags that could narrow over time as tools become more familiar and implementation practices stabilize. The absence of strong evidence for downstream equity gains in generative AI should not be read as evidence of no possible benefit; it reflects a field in which implementation evaluation has not yet kept pace with deployment. The evidence from targeted screening programs in Dominica, Indonesia, and China is genuinely promising. However, only 6% of surgical clinical decision-support studies reported any equity analysis (20), and subgroup auditing remains the exception rather than the rule. Robust equity outcomes are unlikely to follow from deployment volume alone without the structural conditions identified throughout this review.

### Relationship to prior reviews

This review aligns with, but also differs from, recent syntheses in several ways. Berdahl et al. (3) provide an important lifecycle map of health equity issues and mitigation strategies; the present review complements that work by emphasizing post-2020 implementation evidence and distinguishing infrastructure-dependent access barriers from model-dependent performance barriers. Petretto et al. (4) show that devices, literacy, trust, privacy, and social conditions shape participation in digital care; the current review extends it by more clearly separating telehealth infrastructure from AI-specific systems. Ghanem et al. (5) emphasize governance and implementation concerns in public health AI; the present review adds greater emphasis on clinical and quasi-clinical implementation studies. Kim and Backonja (6) show that digital health equity has many frameworks but no single comprehensive model. This review does not claim to offer a wholly new theory. Instead, it contributes a practical integration of digital divide, technology acceptance, and critical public health perspectives to explain how equity effects are produced in real-world AI-enabled digital health settings.

### Integrated analytical model

**Figure 1:**
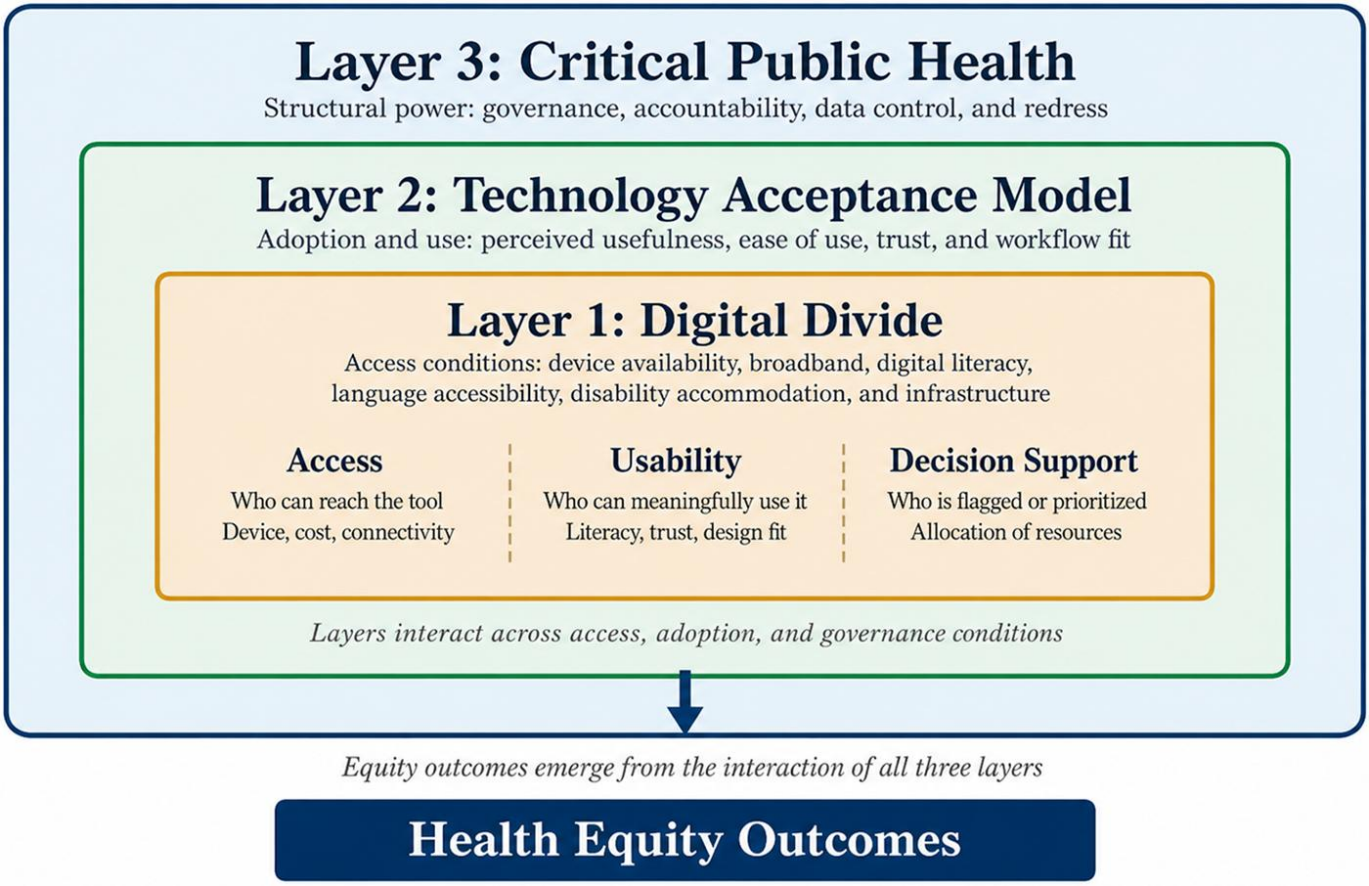
Integrated analytical model of AI-enabled digital health equity. Digital divide conditions shape access to digital health services; technology acceptance model factors influence adoption and sustained use; and critical public health perspectives govern data representation, accountability, and institutional oversight. Health equity outcomes emerge from the interaction of these three layers.

The integrated analytical model used in this review links Digital Divide Theory, the Technology Acceptance Model, and Critical Public Health into a three-layer explanation of how AI-enabled digital health affects equity. Digital Divide Theory explains the structural preconditions of benefit, including broadband access, device availability, digital literacy, language accessibility, disability accommodation, and organizational infrastructure. The Technology Acceptance Model explains uptake and sustained use, showing why patients and clinicians adopt digital tools unevenly depending on perceived usefulness, ease of use, trust, explanation quality, and workflow fit. Critical Public Health explains how power and inequity are embedded in data, institutions, and governance by asking who is represented in training data, which outcomes are optimized, who bears privacy or surveillance burdens, and who has authority over procurement, auditing, override, and redress. Together, these layers suggest that equity effects emerge from the interaction of access, adoption, and governance rather than from technology alone. Figure 1 depicts this integrated analytical model.

### Operational governance and implementation safeguards

Across the corpus, the most consistent lesson is that equity should be governed through procurement, validation, implementation, and maintenance checkpoints rather than appended as a late ethical reflection. This interpretation is consistent with equity-focused implementation models that emphasize embedding equity considerations across the full implementation lifecycle (34). Table 1 translates recurring concerns into operational risk domains, failure modes, safeguards, implementation actions, and illustrative equity indicators that can structure routine decision-making in health system and public-sector deployment contexts.

**Table 1:** Synthesis-derived operational roadmap for equity-sensitive AI implementation in digital health.

Risk Domain	Failure Mode	Safeguard	Implementation Action	Measurement/Indicator
Access Barriers	Digital divide excludes vulnerable populations; geographic, economic, and literacy barriers prevent benefit realization	Universal design; multi-channel access; proactive infrastructure investment	Device lending; low-bandwidth alternatives; digital literacy training; connectivity subsidies; maintain non-digital pathways	Utilization by income, race, age, geography; digital literacy scores; infrastructure coverage gaps
Algorithmic Bias	Training data or optimization criteria encode historical inequities; algorithms underperform for underrepresented groups	Representative training data; diverse teams; subgroup bias testing; interpretable models	Algorithmic impact assessments; disparate-impact audits by race, gender, income; debiasing techniques; bias review boards	Outcome disparities by subgroup; false positive/negative rates; equity audit findings
Privacy and Surveillance	Data collection creates surveillance risks; vulnerable populations avoid services; breaches disproportionately harm marginalized groups	Transparent data governance; minimal data collection; strong security; community-informed consent	Clear data-use policies; privacy impact assessments; differential privacy; opt-out options; community data governance councils	Privacy policy comprehension; data breach incidents; opt-in rates by population; community trust scores
Governance Accountability	Opaque algorithms resist scrutiny; unclear responsibility when AI causes harm; lack of community voice; regulatory gaps	Algorithmic transparency; explainability requirements; participatory governance; regulatory oversight	Algorithmic impact statements; AI ethics committees with community representation; harm reporting systems; equity review in procurement	Algorithm documentation completeness; community governance representation; harm report resolution rates
Workforce Capacity	Clinicians lack AI literacy; technologists lack health equity training; inadequate staffing to monitor equity	Cross-disciplinary training; equity-focused workforce development; adequate human oversight	AI literacy education for clinicians; health equity training for developers; human-in-the-loop requirements; equity analyst positions	Staff training completion; interdisciplinary team composition; human oversight protocols; equity expertise availability

Table distills recurring patterns from the reviewed literature into risk domains, failure modes, safeguards, implementation actions, and illustrative equity indicators to support equity-sensitive AI deployment.

### Equity-safe AI implementation roadmap

The Equity-Safe AI Implementation Roadmap offered in this review is a synthesis-derived, practice-oriented operational heuristic rather than a universal implementation theory or a novel stand-alone framework. It was distilled from recurring patterns identified across the 29 included sources: equitable outcomes in AI-enabled digital health depend on conditions established before deployment, conditions that shape deployment, and conditions that sustain accountability after deployment.

Groom et al. (34) provide a lifecycle-oriented conceptual model for embedding equity across digital health implementation. Table 1 serves a different purpose. Rather than offering another broad implementation model, it organizes the review's findings into a practical risk-domain framework that identifies common failure modes, corresponding safeguards, implementation actions, and illustrative indicators. Its contribution is therefore practical specificity for implementers rather than a new theory of implementation. Groom et al. provide the theoretical justification for why equity must be embedded across the implementation lifecycle; Table 1 provides a practitioner-facing translation for those who must act on that justification in real health systems and public-sector contexts.

### Broader implications for race, proxy, and measurement bias

The concerns identified in this review connect to a broader adjacent literature on inequitable computational and measurement tools in medicine. Work on pulse oximetry bias and race-corrected clinical algorithms shows that seemingly technical instruments can encode assumptions that produce systematically unequal performance across patient groups (22, 23). More recent critical review work further argues that fairness debates in clinical and population-health algorithms extend beyond explicit race variables to unequal decision rates, unequal error rates, and biased target variables (24). These adjacent literatures reinforce the larger point that subgroup validation and accountability cannot be treated as optional add-ons in equity-sensitive deployment contexts.

### Scope and transferability

The findings are most directly applicable to health systems where digital infrastructure already exists but remains unevenly distributed. Evidence from higher-resource settings is stronger on access disparities, uptake patterns, subgroup auditing, and workflow design. Evidence from lower-resource settings is stronger on feasibility, acceptability, and infrastructure dependence. What transfers across settings is not a single model, but a principle: equity depends on local fit, local validation, and local governance.

## Limitations

This review has six main limitations. First, it is a narrative evidence synthesis and therefore cannot provide pooled effect estimates across heterogeneous interventions and settings. Second, the corpus was primarily screened by a single author; a second reviewer independently assessed a sample of 12 full-text decisions with 100% agreement, but residual selection bias remains possible, particularly toward studies using explicit equity framing and away from technically oriented or fast-moving generative AI implementation studies. Third, a formal risk-of-bias tool was not applied; interpretive weighting improves transparency but does not replace formal appraisal. Fourth, the evidence base remained concentrated in the United States and other higher-resource settings; Global South evidence was thinner and less likely to include long-term outcome evaluation. Fifth, empirical studies that directly measured disparity reduction remained limited; this review is therefore stronger at identifying implementation conditions than at estimating the net causal effect of AI on health disparities. Sixth, publication bias likely affects the available evidence base, potentially making beneficial outcomes more visible than null findings or equity failures. These limitations do not invalidate the main conclusions. The strongest findings rest on repeated cross-study patterns: digital access conditions remain unequally distributed, subgroup auditing remains uncommon, and governance capacity strongly shapes whether AI-enabled digital health distributes benefits equitably.

## Future research

Future research should become more specific and more accountable. Implementation studies should routinely report subgroup performance and subgroup benefit distribution rather than only average model accuracy or average satisfaction. More comparative studies are needed to examine whether AI-enabled digital health changes access or outcomes differently across race, language, income, age, disability status, insurance status, and rurality. Empirical work on generative AI should evaluate real-world effects on translation, patient communication, documentation burden, and diagnostic representation in diverse populations. Lower-resource settings need more locally validated implementation studies rather than assumptions of transferability from high-income development contexts. Governance research should evaluate which oversight structures—including procurement rules, audit requirements, community advisory processes, and post-deployment monitoring systems—actually improve equity outcomes in practice.

## Conclusion

Post-pandemic evidence does not support broad claims that AI has already produced major health equity gains. It supports a more specific conclusion: AI-enabled digital health may help address selected access, triage, and specialist-capacity barriers when implementation is supported by adequate infrastructure, user trust, subgroup validation, and accountable governance. Evidence depth varied substantially across modalities, with stronger support for digital access concerns and subgroup performance risks than for equity-improving effects of generative AI. The most useful shift is from asking whether AI is equitable in the abstract to asking whether the surrounding implementation system is equitable in practice. The integrated analytical model linking Digital Divide Theory, the Technology Acceptance Model, and Critical Public Health provides a structured interpretive framework for future implementation research on AI and health equity. The Equity-Safe AI Implementation Roadmap translates recurring evidence patterns into staged operational checkpoints that health systems can apply across procurement, validation, implementation, and post-deployment monitoring to treat equity as a continuous governance requirement rather than an assumed outcome.
