# Supplementary material for: Artificial intelligence and digital health equity: a post-pandemic evidence synthesis and implementation safeguards framework
Source: Front Digit Health. 2026 May 18;8:1785700. doi: 10.3389/fdgth.2026.1785700 (PMC13223092; doi:10.3389/fdgth.2026.1785700)
Supplement: Supplementary file 1 [file Table1.docx]

**Supplementary Table S1. Evidence inventory of the 28 studies included in the final evidence corpus.**

The table below summarizes the 28 sources included in the final corpus. Sources cited only for peripheral contextual support outside the final corpus are not listed here.

| Author/Year | Study Design | Country/Setting | Population | AI/Digital Tool | Equity-Relevant Outcome | Corpus Type |
| --- | --- | --- | --- | --- | --- | --- |
| Lam et al. (2020) | Cross-sectional analysis | United States | Older adults | Telemedicine readiness assessment | Estimated telemedicine unreadiness due to functional and technological barriers | Empirical |
| Adepoju et al. (2024) | Cross-sectional survey | United States | Lower-income urban adults | RPM, medical apps, wearables | High smartphone ownership but low RPM/app/wearable use; racial differences in RPM use | Empirical |
| Blount et al. (2023) | Mixed-methods study | United States | Primary care clinicians in underserved communities | Digital health tools | High clinician adoption but persistent patient-side access barriers | Empirical |
| Kemp et al. (2023) | Prospective clinical validation study | Dominica | Adults with diabetes in screening program | Smartphone-based AI diabetic retinopathy screening | Reasonable sensitivity and specificity in real-world screening | Empirical |
| Harsono et al. (2022) | Diagnostic development study | Indonesia | Women undergoing VIA screening | Smartphone-based AI VIA application | High sensitivity, specificity, and accuracy for cervical screening support | Empirical |
| Gu et al. (2023) | Real-world multicentre cross-sectional study | China | Primary care participants | AI fundus disease screening system | High accuracy and specificity with variable sensitivity by condition | Empirical |
| Nakisige et al. (2023) | Diagnostic comparison study | Cervical screening context | Healthcare workers, experts, algorithm | AI-assisted cervical visual inspection | Algorithm comparable to healthcare workers and below expert performance | Empirical |
| Mooney et al. (2024) | Clinical intervention study | United States | Patients with cancer | Automated digital symptom monitoring and management | High engagement and lower symptom burden than usual care across most subgroups | Empirical |
| Lo-Ciganic et al. (2021) | Machine-learning prognostic study | United States | Medicaid beneficiaries | Overdose risk prediction model | Social-context data modestly improved prediction performance | Empirical |
| Pandey et al. (2021) | Qualitative study | Canada | Immigrants and healthcare providers | Language access context | Limited English proficiency delayed access and weakened care relationships | Empirical |
| Sachdeva et al. (2024) | Qualitative study | Cameroon | Women aged 30–49 | AI-assisted cervical screening support | Acceptability shaped by privacy, usefulness, trust, and explanation quality | Empirical |
| Joerg et al. (2025) | Experimental study | Multi-platform | AI-generated dermatologic images | Generative image AI | Light-skin overrepresentation and poor diagnostic identifiability | Empirical |
| Berdahl et al. (2023) | Scoping review | Multiple | AI and health equity literature | AI lifecycle issues and strategies | Identified 18 equity issues and 15 strategies | Review |
| Petretto et al. (2024) | Scoping review | Multiple | Telemedicine and e-health literature | Telemedicine/e-health | Barriers related to literacy, privacy, language, and access | Review |
| Ghanem et al. (2025) | Rapid narrative review | Canada | AI in public health literature | Public health AI | Bias, accessibility, governance, and transparency concerns | Review |
| Kim & Backonja (2025) | Scoping review | Multiple | Digital health equity frameworks | Frameworks and concepts | Found 42 frameworks and no single comprehensive model | Review |
| Fliorent et al. (2024) | Review | Multiple | Dermatology AI programs | Dermatology AI tools | Underrepresentation of skin of color in AI dermatology evidence | Review |
| Paulus & Kent (2020) | Conceptual analysis | Clinical prediction contexts | Not applicable | Clinical prediction models | Clarified fairness versus bias in algorithmic prediction | Framework |
| Richardson et al. (2022) | Framework paper | Digital health systems | Digital health users and systems | Framework for digital health equity | Multilevel digital determinants of health | Framework |
| Groom et al. (2024) | Framework paper | Digital health implementation | Researchers and implementers | DH-EquIR model | Equity should be planned across implementation phases | Framework |
| Lyles et al. (2021) | Perspective / commentary | Healthcare delivery systems | Digital health systems | Digital health | Digital access and skills as foundational determinants | Framework |
| Alami et al. (2020) | Conceptual/policy analysis | LMICs | AI implementation contexts | AI in healthcare | Outlined responsible and inclusive AI building blocks for LMICs | Governance/Policy |
| Townsend et al. (2023) | Regulatory mapping study | Africa | 12-country regulatory landscape | AI in healthcare regulation | Fragmented regulation mediated through adjacent legal regimes | Governance/Policy |
| Wagner et al. (2024) | Governance analysis | Public health contexts | AI governance contexts | AI governance | Public health perspectives can strengthen AI governance | Governance/Policy |
| Hernandez-Boussard et al. (2023) | Policy/analysis article | United States | Clinical decision-making contexts | Race-based clinical algorithms | Race-based tools can produce inequitable recommendations | Governance/Policy |
| López et al. (2022) | Review | LMICs | AI health ecosystem literature | AI implementation challenges in LMICs | Identified 40 challenges and 89 recommendations | Review |
| Ingraham et al. (2022) | Scoping review | United States (clinical surgery settings) | Surgical patients / general clinical populations | Clinical decision support tools | Only 5 of 84 studies (6%) reported equity analysis | Review |
| Ortega et al. (2021) | Conceptual analysis | United States | Patients with limited English proficiency | Language-access tools | Structural language accommodation needed for equitable care | Framework |
